# Effects of a stress management program on third year medical students' anxiety depression and somatization

**DOI:** 10.15694/mep.2017.000044

**Published:** 2017-03-06

**Authors:** Angele McGrady, Denis Lynch, Julie Brennan, Kary Whearty

**Affiliations:** 1 University of Toledo Medical Center, Toledo, USA

**Keywords:** medical students, stress management, anxiety, depression

## Abstract

This article was migrated. The article was marked as recommended.

**Background:** Transition from the medical school classroom to the clinical training years requires students to adapt in many ways. Schedules are more variable, with longer clinic hours and travel to affiliated hospitals. Students are also faced with emotional needs of patients coincident with meeting demands from attending physicians. The prevalence of anxiety, depression and overall distress increases during the four years of medical school and particularly during difficult transitions.

**Methods:** Forty medical students entering their first clinical year enrolled in a two session stress management program focused on mindfulness and coping strategies. Sessions were interactive, conducted by a psychologist, social worker and a counselor and comprised evidenced based components.

**Results:** Twenty nine students completed the program. Baseline comparisons between dropouts and eventual completers showed that dropouts were more likely to screen positive for depression, anxiety and somatic tendencies. Program completers evidenced short term increased knowledge about mindfulness and coping and demonstrated significant decreases in anxiety and somatization at the end of the program.

**Conclusion:** Though scheduling of any additional programs during the clinical years of medical school presents significant challenges, students who complete such a program sustain important benefits and evaluate the program positively.

## Background

The stressors related to the clinical years of medical school have been documented previously ( Compton, Carrera, & Frank, 2008; Schwenk, Davis, & Wimsatt, 2010). While the first two years are classroom and laboratory based, the third year emphasizes learning in actual patient care situations. Frequent changes in the academic schedule, more responsibilities and away rotations limit time with friends. Ethical dilemmas and caring for terminally ill patients can all add to the emotional burden on students.

Symptoms of anxiety and depression often increase throughout the medical school years ( Compton, Carrera, & Frank, 2008). While most students are able to cope with these challenges, some demonstrate impaired functioning and well-being ( Coentre & Figueira, 2016). Within this context, programs to provide added support to medical students have been designed. A comprehensive program comprising small and large group wellness programming during each year of medical school received excellent student evaluations ( Drolet & Rodgers, 2010). In an effort to improve first year medical students’ ability to cope with stress, an eight session intervention including such topics as relaxation, coping skills, mindfulness and maintaining balance was offered. Students sustained significant decreases in depression at the end of the program compared to a wait list control group ( McGrady, Brennan, Lynch, & Whearty, 2012). A subgroup of first year students identified as high risk were more compliant to recommendations for relaxation practice and significantly reduced scores on anxiety inventories ( Brennan, McGrady, Lynch, Schaefer, & Whearty, 2016). Herein, we describe a program of stress management for third year medical students and its effects on anxiety, depression, and somatization symptoms. Specifically, our hypothesis was that the intervention would be helpful to students in reducing anxiety, depression and somatic preoccupation.

## Method

**Participants:** Students entering their clinical year of medical school at a medium sized medical school were potential participants. The program was offered to approximately 300 students.

**Procedure:** The study was approved by the IRB at the institution and all participants signed the consent form.

**Recruitment:** Students were required to attend a week long orientation covering a variety of topics, including Student Wellness. During one of the presentations students were presented with the opportunity to participate in a voluntary stress management program. Participants completed a demographic questionnaire, and standardized tests assessing anxiety, depressive symptoms, and tendency to somatize. Instruments: The Patient Health Questionnaire-9 (PHQ-9) ( Kroenke, Spitzer, & Williams, 2001), the Generalized Anxiety Disorder (GAD7), ( Spitzer, Kroenke, Williams, & Lowe, 2006), and the Patient Health Questionnaire-15 (PHQ-15) ( Kroenke, Spitzer, & Williams, 2002) were administered at baseline and after the two sessions.

**Intervention:** Two sessions were offered during the academic year: one on mindfulness during the Family Medicine rotation and one on coping skills during the Psychiatry rotation. Faculty members from both departments were supportive of the program. A total of about 150 minutes was spent in face to face contact between facilitator and students. Participants were encouraged to practice the techniques learned during the sessions, ideally on a daily basis.

The session held during Family Medicine educated participants on Mindfulness and centered on being fully present in the moment in a nonjudgmental manner. Emphasis was placed on improving awareness when the mind gets distracted during patient interviews or lectures. The presenter engaged participants by asking open ended questions, providing case scenarios, and using reflection on past situations. This session ended with 10 minutes of mindful practice ( Krasner, Epstein, Beckman, Suchman, Chapman, Mooney, & Quill, 2009; Fortney, Luchterband, Zakletskaia, Zgierska, & Rakel, 2013). Students completed a rating scale indicating knowledge of mindfulness before and after this session.

The session held during Psychiatry focused on positive coping ( Seligman, Steen, Park, & Peterson, 2005). With the use of a short questionnaire, students identified their most common coping style and the situations where that coping method was most and least likely to be successful. Empathic listening while maintaining boundaries with patients was discussed and role played. Students identified 3 things for which they were grateful on that day. They identified a few healthy pleasures, whether large or small and were recommended to enjoy what they had listed as often as appropriate. This session ended with a brief relaxation exercise, specifically slow paced breathing ( Davis, Eschelman, & McKay, 2008). Students completed a rating scale indicating perceived sense of relaxation before and after the exercise.

**Analysis:** Data were analyzed with SPSS. Descriptive statistics were generated. ANOVA compared baseline information on participants who dropped out with those who completed the program and to compare baseline and post intervention data for the completers. There is missing data since the numbers of students completing the questionnaires varied. Pre and post session data on knowledge of mindfulness and experience of relaxation was collapsed across all participants and all sessions. Since the direction of the differences was predicted in our hypotheses, one-tailed tests were used to determine significance.

## Results

Sixty three students signed the consent form. Forty students (27 women and 13 men) of mean age 25 years completed the initial assessments. Most (26) were single, but 13 were either married or living with a partner. Nine students screened positive on the PHQ-15, 5 screened positive on the GAD-7 and 7 on the PHQ-9.

Twenty nine students attended both sessions (completers); eleven did not. The dropout rate for those who screened positive on the PHQ-9, or the PHQ-15 or the GAD-7 was above 50%.

Table 1 details the comparison between the PHQ-15, GAD-7, and PHQ-9 scores in completers and dropouts. Dropouts were found to have significantly higher scores on the PHQ-9 (p > 0.02) and GAD-7 (p < 0.03) ( Table 1a). Completers were compared on baseline and post two sessions on GAD-7, PHQ-9 and PHQ-15. In the program completers, significant decreases in GAD-7 scores (p< 0.025) and PHQ-15 (p < 0.036) were observed ( Table 1b). Furthermore, students demonstrated significant increases in knowledge of mindfulness and greater perceived relaxation following the sessions (mindfulness: paired t-test; t= -8.16 p <0.0001 and relaxation: t = -7.43 p < 0 .0001). Student evaluations of the program were positive, emphasizing the practical applications that were discussed and the building of relaxation and mindfulness skills.

## Discussion

The percentage of participants in this study who screened positive on the assessment instruments (between 13% and 23%) highlights the fact that some students may need extra support services during the clinical years of medical school. A similar percentage of positive screens have reported in other groups of medical students entering their first clinical year ( Brennan, McGrady, Whearty, Lynch, Rapport, & Schaefer, 2012; Roberts, 2010). Importantly, it has been reported that distress correlates with more negative patient interactions and less empathy by medical students engaged in clinical rotations ( Hojat, Vergare, Maxwell, Brainard, Herrine, Isenberg, & Gonnella, 2009).

Unfortunately, students who screened positive or who had significantly higher scores on anxiety, depression and somatization were more likely to drop out of the program. It may be that the relatively high levels of anxiety and depression signaled worry about academic performance so they decided to spend the time studying. Students may also have recognized that the program was not sufficient to deal with their level of distress. Our findings support those of similar studies utilizing interventions of different intensity, length and outcome measures. For example, Phang et al (2015) found short term improvements in perceived distress and greater sense of self efficacy in medical student participants compared to a control group. Further research is needed to determine if the post program results continue long term.

## Conclusion

Relatively brief interventions can produce observable improvements in student ratings of distress and students rated the program positively. Mindfulness, stress management and coping seem to be important components ( van Dijk, Lucassen, P.L., & Speckens, A.E., 2015). Noteworthy is the common difficulty in recruiting and retaining students for any extra programs, particularly students in their clinical years ( Greeson, Toohey, & Pearce, 2015). Interventions must be brief, focused and ideally, integrated into specific clinical rotations. Findings from this study and others suggest that it is worthwhile to continue offering programs for students engaging in clinical rotations, despite the inherent challenges.

## Take Home Messages

• Over 10% of medical students entering their 3rd year screen positive for anxiety and depression.
• Personal distress interferes with effective patient care.
• Brief stress management interventions reduce distress.
• Interventions must be brief, focuse, and must include mindfulness and coping strategies.

## Notes On Contributors

Dr. Angele McGrady received her B.S. from Chestnut Hill College, her Masters in Physiology from Michigan State University, her Ph.D. in Biology and her M. Ed in Counseling from the University of Toledo. She is a licensed counselor and a certified provider of biofeedback. Currently Dr. McGrady is a professor in the Department of Psychiatry at the University of Toledo Medical Center (UTMC), where she also maintains a practice in counseling and biofeedback. Dr. McGrady’s professional activities include: Past President of the Association for Applied Psychophysiology and Biofeedback and past Associate Editor of “Applied Psychophysiology and Biofeedback”. Dr. McGrady has experience in teaching: medical, nursing, physician assistant, graduate students and residents. She designed wellness programs for medical students, residents and more recently college level athletes. Her resume lists 85 peer reviewed articles and book chapters. Her book “Pathways to Illness, Pathways to Health” with Donald Moss, Ph.D. was released in 2013 by Springer.

Dr. Julie Brennan received her B.S. in Dietetids and Psychology from University of Dayton - and her Masters of Arts and Ph.D. in Philosophy from The Ohio State University. Currently Dr. Brennan is an Assistant Professor in Family Medicine at the University of Toledo Medical Center. Dr. Brennan’s research interests include wellness, health behavior change, eating disorders, obesity, depression and anxiety.

Dr. Denis Lynch is Professor Emeritus of Psychology in Family Medicine and Psychiatry at the University of Toledo Medical Center.

Kary Whearty is a licensed social worker who has participated in wellness programs for the past 10 years. These wellness initiatives have benefited medical students, patients, and healthcare workers.

## Appendices

**Table 1a.** Comparison of dropouts and eventual completers at baseline

	PHQ-15	PHQ-9 *	GAD-7 **
**Completers (27)**	6.4 (3.7)	3.8 (3.8)	4.4 (3.6)
**Drop Outs (10)**	8.5 (4.2)	7.4 (4.6)	7.3 (3.3)

ANOVA * *F*(1,34) = 5.8 *p*=.021, ** *F*(1,35) =4.9 *p*=.033

**Table 1b.** Comparison of Measures of Distress at Baseline and After the Intervention

	#PHQ-15 (n-19)	PHQ-9 (n=17)	##GAD-7 (n=19)
**Baseline**	6.7 (3.8)	4.3 (4.1)	4.9 (3.8)
**Post Intervention**	5.3 (4.1)	3.6 (3.6)	3.3 (3.2)

Paired t-test: # t= 1.91 p =.036; ## t= 2.07 p =.025

## References

[ref1] Brennan J. McGrady A. Lynch D. Schaefer P. & Whearty K. (2016). A stress management program for higher risk medical students: Preliminary findings. Applied Psychophysiology and Biofeedback. 41(3),301–305. 10.1007/s10484-016-9333-1 26969177

[ref2] Brennan J. McGrady A. Whearty K. Lynch D. Rapport D. & Schaefer P. (2012). Emotional status of third year medical students and their responses to a brief intervention. Annals of Behavioral Science and Medical Education. 18(2),3–7.

[ref3] Coentre R. & Figueira M. (2016). Depression and suicidal behavior in medical students: A systematic review. Current Psychiatry Review. 11(2),86–101. 10.2174/1573400510666140807005141

[ref4] Compton M. Carrera A. & Frank E. (2008). Stress and depressive symptoms/dysphoria among US medical students. Journal of Nervous and Mental Disease. 196(12),891–897. 10.1097/NMD.0b013e3181924d03 19077856

[ref5] Davis M. Eschelman E. R. & McKay M. (2008). The relaxation and stress reduction workbook. 6th Ed. Oak Park, California: New Harbinger Publication, Inc.

[ref6] Drolet B. C. & Rodgers S. (2010). A comprehensive medical student wellness program-design and implementation at Vanderbilt School of Medicine. Academic Medicine. 85(1),103–110. 10.1097/ACM.0b013e3181c46963 20042835

[ref7] Fortney L. Luchterband C. Zakletskaia L. Zgierska A. & Rakel D. (2013). Abbreviated mindfulness intervention for job satisfaction, quality of life, and compassion in primary care clinicians: a pilot study. Annals of Family Medicine. 11(5),412–420. 10.1370/afm.1511 24019272 PMC3767709

[ref8] Greeson J. M. Toohey M. J. & Pearce M.J. (2015). An adapted, four-week mind-body skills group for medical students: reducing stress, increasing mindfulness, and enhancing self-care. Explore. 11,186–192. 10.1016/j.explore.2015.02.003 25792145

[ref9] Hojat M. Vergare M. J. Maxwell K. Brainard G. Herrine S. K. Isenberg G. A. & Gonnella J. S. (2009). The devil is in the third year: a longitudinal study of erosion of empathy in medical school. Academic Medicine. 84,1182–1191. 10.1097/ACM.0b013e3181b17e55 19707055

[ref10] Krasner M. Epstein R. Beckman H. Suchman A. Chapman B. Mooney C. & Quill T. (2009). Association of an educational program in mindful communication with burnout, empathy, and attitudes among primary care physicians. JAMA. 302(12),1284–1293. 10.1001/jama.2009.1384 19773563

[ref11] Kroenke K. Spitzer R. & Williams J. (2001). The PHQ9: Validity of a brief depression severity measure. Journal of General Internal Medicine. 16(9),606–613. 10.1046/j.1525-1497.2001.016009606.x 11556941 PMC1495268

[ref12] Kroenke K. Spitzer R. & Williams J. (2002). The PHQ15: Validity of a new measure for evaluating somatic symptom severity. Psychosomatic Medicine. 64(2),258–266. 10.1097/00006842-200203000-00008 11914441

[ref13] McGrady A. Brennan J. Lynch D. & Whearty K. (2012). A wellness program for first year medical students. Applied Psychophysiology and Biofeedback. 37,253–260. 10.1007/s10484-012-9198-x 22699926

[ref14] Phang C. K. Mukhtar F. Ibrahim N. Keng S. L. & Mohd Sidik S. (2015). Effects of a brief mindfulness-based intervention program for stress management among medical students: the mindful-gym randomized controlled study. Advances in Health Science Education: Theory & Practice. 20(5),1115–1134. 10.1007/s10459-015-9591-3 25697124

[ref15] Roberts L.W. (2010). Understanding depression and distress among medical students. JAMA. 304(11),1231–1233. 10.1001/jama.2010.1347 20841539

[ref16] Schwenk T. Davis L. & Wimsatt L. (2010). A program for reducing depressive symptoms and suicidal ideation in medical students. JAMA. 304(11),1181–1190. 20841531 10.1001/jama.2010.1300

[ref17] Seligman M. E. P. Steen T. A. Park N. & Peterson C. (2005). Positive psychology progress: Empirical validation of interventions. American Psychology. 60,410–421. 10.1037/0003-066X.60.5.410 16045394

[ref18] Spitzer R. L. Kroenke K. Williams J. B. & Lowe B. (2006). A brief measure for assessing generalized anxiety disorder: the GAD7. Archives of Internal Medicine. 166,1092–1097. 10.1001/archinte.166.10.1092 16717171

[ref19] van Dijk I. Lucassen P. L. & Speckens A. E. (2015). Mindfulness training for medical students in their clinical clerkships: Two cross-sectional studies exploring interest and participation. BMC Medical Education. 15,24. 10.1186/s12909-015-0302-9 25888726 PMC4348100

