# Supplementary material for: Effects of a stress management program on third year medical students' anxiety depression and somatization
Source: MedEdPublish (2016). 2017 Mar 6;6:44. [Version 1] doi: 10.15694/mep.2017.000044 (PMC10885240; doi:10.15694/mep.2017.000044)
Supplement: Peer review history

# Reviewer report: Reviewer response for version 1

**DOI:** 10.21956/mep.19453.r28656

**Published:** 2017-03-07

**Authors:** Susan Van Schalkwyk

**Affiliations:** 1 Stellenbosch University

This review has been migrated. The reviewer awarded 3 stars out of 5 This article describes an aspect of medical education that remains a particular challenge and one that requires our attention. The effects of anxiety and depression among the student population is cause for concern, particularly in countries where as graduates they will be required to work in under-resourced contexts. The nature of the intervention, therefore, appears to be one that has merit as it seeks to support students in dealing with some of the challenges resulting from the stress of moving from classroom based teaching and learning to the clinical context. The article is quite short and therefore the reader is expected to fill in the gaps which is not always useful. Reference is made to several different instruments and it is not always clear what was used when. Also, it would have been interesting to have had some rationale for the instruments that were selected – why these and not others?I also believe that the article would have been strengthened by considering some of the findings more critically. The students who participated in the programme were self-selected. Given that this appears to have been a relatively small sub-set of the full group (40/300??), it could have been interesting to reflect on why these students elected to follow this option. Why did 63 initially sign up and only 40 participate? A comment about academic challenges was noted as a possible reason for only 29 of the 40 completing, but this seems to be pure conjecture as there is no evidence for this. Similarly, the conclusion about the nature of such interventions (brief, focused, integrated) all ring true, but cannot be offered as conclusions from the study. Perhaps some qualitative work as a next step could be of value?

Reviewer Expertise:

NA

No decision status is available

# Reviewer report: Reviewer response for version 1

**DOI:** 10.21956/mep.19453.r28658

**Published:** 2017-03-06

**Authors:** Trevor Gibbs

**Affiliations:** 1 AMEE

This review has been migrated. The reviewer awarded 3 stars out of 5 An interesting paper about a very difficult but all to common a problem amongst, I would imagine, all tertiary students. The results were deemed positive to the extent that the authors supported the inclusion of similarly structured courses within other curricula.Whilst not wishing to disagree with the authors as to the value of this intervention, I do wonder if such an activity brings in a false sense of security. I think that the attrition rate of students wishing to take part in such an activity needs exploration, particularly since the authors found that anxiety / depression / somatisation scores were higher in this group. Certainly I would agree with my co-reviewers that a longer term follow up of all participants and non-participants is indicated. With this in mind I am not sure that I can agree with the take home messages of the authors.

Reviewer Expertise:

NA

No decision status is available

# Reviewer report: Reviewer response for version 1

**DOI:** 10.21956/mep.19453.r28659

**Published:** 2017-03-06

**Authors:** Manish Khazane

**Affiliations:** 1 SRM UNIVERSITY,RAMAPURAM,CHENNAI.

This review has been migrated. The reviewer awarded 3 stars out of 5 Angele McGrady et al,2017 aimed to assess the anxiety,depression and somatic preoccupation of the medical students entering into the clincial rotation phase of their medical schools.Authors emphazied on topics such as Coping skills,mindfulness and maintaining balance during this phase.Two sessions included, mindfulness during the family medicine rotation and one on coping skills during the Psychiatry rotation. Post intervention sessions, it was found that students demonstrated significant increase in the knowledge of mindfulness and relaxation.Although the study finds the level of improvement due to the concerned intervention, study aptly excludes students with higher anxiety/distress levels who should be the main targets to be worked upon. Although there is a definite agreement as stated by the author that interventions should be brief,focused and integrated into specific clinical rotations. Further focus should be laid upon finding and treating those students who are battling with anxiety,depression and sufficient support should be enabled for them.

Reviewer Expertise:

NA

No decision status is available

# Reviewer report: Reviewer response for version 1

**DOI:** 10.21956/mep.19453.r28657

**Published:** 2017-03-06

**Authors:** Julie Hunt

**Affiliations:** 1 Lincoln Memorial University

This review has been migrated. The reviewer awarded 4 stars out of 5 The mental health of medical students and medical providers is a critical area where medicine stands to improve. This article describes a two-session intervention for medical students and evaluates its impact on mental health. This intervention is concise and incorporated into an existing clinical rotation, both of which make it more appealing to students and educators. I agree with the authors that a follow-up study evaluating lasting impact would be helpful in justifying the value of the intervention and perhaps suggesting how often during a student's education this intervention should be offered. Disturbingly, the drop-outs were more in need of mental health intervention than the completers. The authors suggest a few reasons for this. Interviewing or surveying those who dropped out about their reasons could have elucidated that and provided valuable information to the authors and readers about how to avoid this disturbing trend. Overall, this is a good initial evaluation of an intervention that appears reasonable in the time and effort required to deliver it.

Reviewer Expertise:

NA

No decision status is available
